# Capturing ultrafast photoinduced local structural distortions of BiFeO_3_

**DOI:** 10.1038/srep15098

**Published:** 2015-10-14

**Authors:** Haidan Wen, Michel Sassi, Zhenlin Luo, Carolina Adamo, Darrell G. Schlom, Kevin M. Rosso, Xiaoyi Zhang

**Affiliations:** 1 X-ray Science Division, Argonne National Laboratory, Argonne, Illinois 60439, USA; 2 Physical Sciences Division, Pacific Northwest National Laboratory, Richland, WA 99354, USA; 3 National Synchrotron Radiation Laboratory, University of Science and Technology of China, Hefei, 230029, China; 4 Department of Materials Science and Engineering, Cornell University, Ithaca, New York 14853, USA; 5 Kavli Institute at Cornell for Nanoscale Science, Ithaca, New York 14853, USA

## Abstract

The interaction of light with materials is an intensively studied research forefront, in which the coupling of radiation energy to selective degrees of freedom offers contact-free tuning of functionalities on ultrafast time scales. Capturing the fundamental processes and understanding the mechanism of photoinduced structural rearrangement are essential to applications such as photo-active actuators and efficient photovoltaic devices. Using ultrafast x-ray absorption spectroscopy aided by density functional theory calculations, we reveal the local structural arrangement around the transition metal atom in a unit cell of the photoferroelectric archetype BiFeO_3_ film. The out-of-plane elongation of the unit cell is accompanied by the in-plane shrinkage with minimal change of interaxial lattice angles upon photoexcitation. This anisotropic elastic deformation of the unit cell is driven by localized electric field as a result of photoinduced charge separation, in contrast to a global lattice constant increase and lattice angle variations as a result of heating. The finding of a photoinduced elastic unit cell deformation elucidates a microscopic picture of photocarrier-mediated non-equilibrium processes in polar materials.

Photoinduced phenomena in polar materials such as ferroelectrics and multiferroics have attracted great interest because of the fundamental physics underlying energy exchange among multiple degrees of freedom and the technological relevance to photovoltaic and information processing devices^1^. The intrinsic symmetry breaking due to spontaneously ordered ferroelectric polarization interacts with light-generated charge carriers, which in turn influence the electronic and structural properties of the materials. In the multiferroic archetype BiFeO_3_ (BFO), the charge separation and transfer upon optical excitation can induce large, above-band-gap photovoltaic voltages^2^ and sizable changes in crystal dimensions^3^,^4^. Recent studies using ultrafast x-ray and optical probes showed that a macroscopic out-of-plane strain^5^ and shear strain^6^ can be generated upon optical excitation. These processes can occur locally and give rise to an inhomogeneous strain profile due to trapped photo-carriers^7^. However, the local structural configuration around a transition metal atom upon photoexcitation, including interaxial lattice angle, bond length, oxygen octahedron rotation, are challenging to measure directly. These information is crucial to the understanding the microscopic processes of photostriction in polar materials^8^.

Although time-resolved x-ray absorption spectroscopy (TR-XAS) in the hard x-ray region has been widely used to probe photoinduced changes in local geometric structures and electronic configurations of molecular systems^9^,^10^,^11^, its application to complex condensed matter systems is just starting to be explored^12^,^13^. Using time-resolved hard x-ray spectroscopy measurements and density functional theory (DFT) calculations, we show the complex atomic structural dynamics in a pseudocubic unit cell following optical excitation. First, the unit cell experiences a uniaxial out-of-plane expansion, accompanying an elastic in-plane lattice contraction, as a result of localized interaction of remnant polarization and trapped charge carriers. Next, the lattice angles remain constant upon photocarrier injection, in contrast to a change of these angles upon heating. Variations in the Fe-O average bond distances and oxygen octahedral tilt angles are negligible, insensitive to optical excitation or thermal heating. Finally, the photoinduced x-ray absorption changes, which provides a fingerprint for the local structural configuration around Fe, relax at the same rate as that of the ensemble averaged strain as measured by time-resolved x-ray diffraction (TR-XRD), indicating the same electronic origin resulting from the interplay of ferroelectric polarization and photocarriers. The optical excitation leads to microscopically localized structural changes which cannot be achieved by thermal heating, providing a unique ultrafast control scheme for ferroelectrics.

## Results

### Time-resolved x-ray absorption spectroscopy

**Figure 1:**
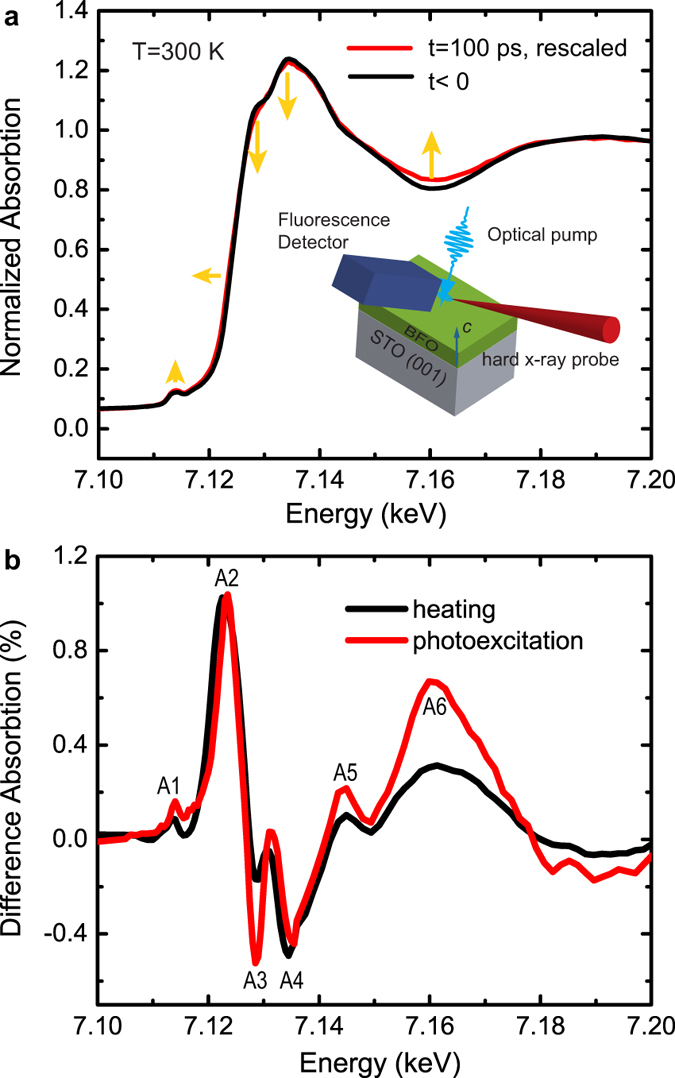
(**a**) The absorption spectra at Fe *K*-edge before (t < 0) and after (t = 100 ps) the 400 nm laser excitation measured at room temperature. To better illustrate the laser induced spectral changes, the difference as shown in the red curve comparing to the black curve is multiplied by a factor of 5. The arrows indicate the changes of spectral features. The inset shows the schematic of the time-resolved XAS setup. (**b**) The difference absorption spectra at Fe *K*-edge upon photoexcitation (t = 100 ps) and heating. The difference spectrum upon heating was obtained by subtracting the absorption spectrum measured at 300 K from the spectrum measured at 550 K. The amplitude of thermally induced spectrum (black) is reduced by a factor of 7.4 to normalize to the amplitude of A2 of the photoinduced spectrum.

A 35 nm BFO film was grown on SrTiO_3_ (001) by reactive molecular beam epitaxy^14^. The time-resolved x-ray absorption spectra at the Fe *K*-edge were collected in fluorescence mode at the 11ID-D beamline of the Advanced Photon Source (APS) (Methods). Figure 1a shows the Fe *K*-edge x-ray absorption near edge structure (XANES) before (t < 0) and after (t = 100 ps) excitation by a 400 nm, 100 fs optical pulse, with an absorbed fluence of 1.4 mJ/cm^2^. The penetration depth of 400 nm light in BFO is comparable to the film thickness^14^, giving rise to a variation of excitation fluence along the depth of the film. This fluence variation does not significantly complicate data analysis since the photoinduced effect is linearly proportional to the pump laser fluence^5^. The pre-edge peak at 7115 eV corresponds to the 1*s* to 3*d* transition, and the main edge transitions at 7128 eV and 7135 eV correspond to the transition from 1*s* to 4*p* unoccupied states. These transitions for the pre- and main-edges have been confirmed by an analysis of the electronic density of states obtained theoretically (see Supplementary Materials Note 2, 3). Features between 7140 eV to 7160 eV originate from multiple-scattering processes. The peak around 7190 eV is mainly due to the single-scattering of the outgoing photoelectrons from the first coordination shell of O atoms.

The difference spectrum following photo-excitation is shown in Fig. 1b. We observed a small increase of the absorption (A1 peak) at 7115 eV, close to that of the pre-edge feature in the XANES spectra. At 7123 eV, the absorption increases noticeably (A2 peak) before the rise of the main *K*-edge upon laser excitation, indicating a small red shift of the absorption edge. This peak is followed by two valleys, labeled A3 and A4, at 7129 eV and 7135 eV, respectively. At higher energies, the absorption increases again, giving rise to peaks at 7145 eV and 7160 eV, labeled as A5 and A6 respectively.

In order to differentiate the electronic and thermal contributions, temperature-dependent x-ray absorption spectra were also measured at 300 and 550 K, below the Néel temperature of BFO. The difference spectrum is shown as a black curve in Fig. 1b. The difference in the thermal expansion coefficients of the film and substrate does not contribute significantly to temperature-dependent XAS measurement, as supported by the agreement of the thin film measurement with the previous report on bulk BFO^15^. Although the overall shape of the difference spectrum is similar to the photoinduced spectrum, the temperature increase induces an uneven decrease of absorption at A3 and A4 and a smaller increase at A6. These features are different from the results upon the optical excitation and will be discussed later.

**Figure 2:**
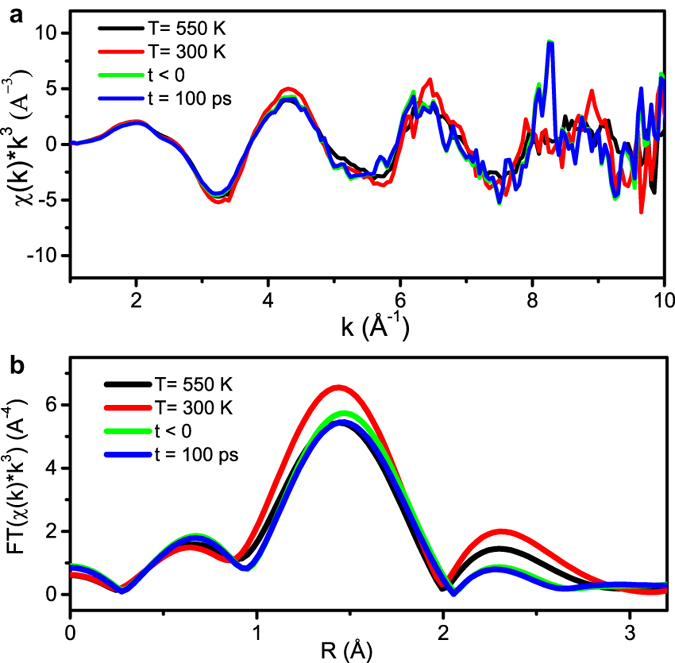
(**a**) Fe *K*-edge EXAFS spectra in *K* space. before (t < 0) and after (t = 100 ps) the laser excitation taken at room temperature. Similar spectra without laser excitation were taken at 300 and 550 K respectively. (**b**) Fourier transformed spectra in *R* space, which are not phase corrected.

To evaluate the average bond distance change, we measured extended x-ray absorption fine structure (EXAFS). Figure 2a,b show the EXAFS spectra before and at 100 ps after optical excitation in *k* space and the magnitude of phase-uncorrected Fourier transformed EXAFS in *R* space. The peaks between 1–2 Å in Fig. 2b are associated with the distance between Fe and its first coordination shell O. To compare with temperature-dependent spectra, the EXAFS spectra at 300 and 550 K were also measured and analyzed respectively. The detailed data analysis similar to refs 10, 16 is given in the Supplementary Materials Note 7. It shows that the average bond Fe-O distance was not affected by either heating or optical excitation within the accuracy of the experimental uncertainty of 0.02 Å. This observation is consistent with the DFT calculation as discussed later. The averaged structural response in domains with different polarization states, electronic and structural configurations, may also contribute to the non-observable changes of bond length.

**Figure 3:**
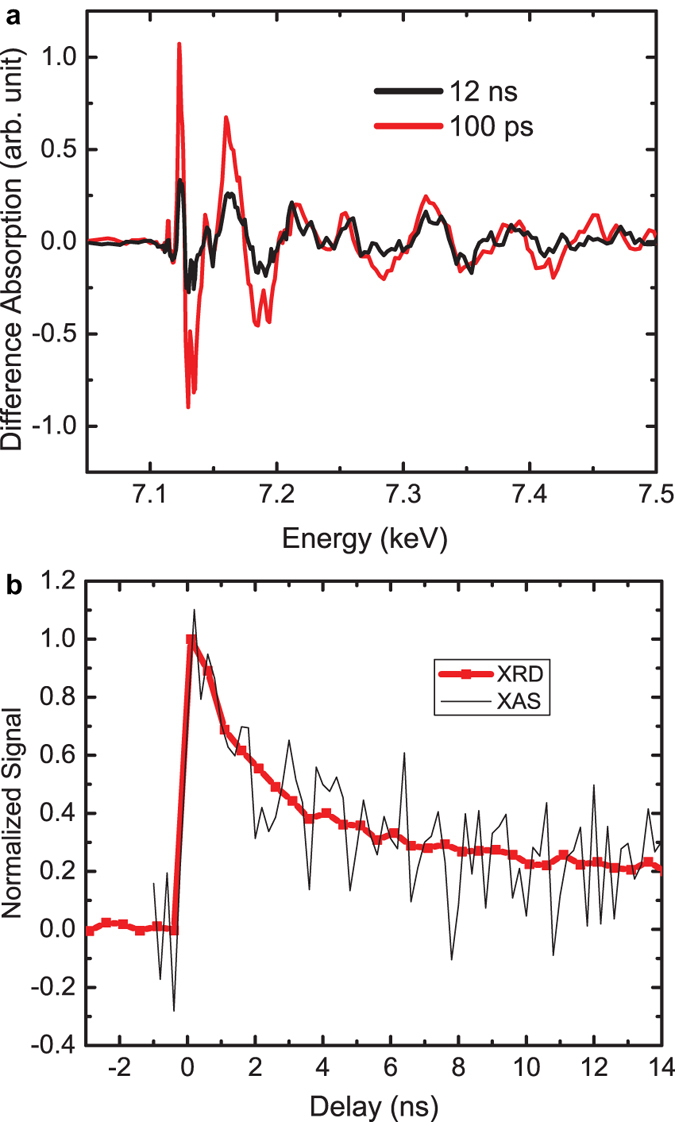
(**a**) The x-ray absorption spectra at delay t = 100 ps and 12 ns. (**b**) The normalized recovery of photoinduced out-of-plane strain measured by TR-XRD (from ref. 5), with an absorbed fluence of 1.9 mJ/cm^2^) and x-ray absorption change at 7123 eV as a function of the pump-probe delay.

The change of the absorption spectrum as a function of time was measured at 100 ps and 12 ns after laser excitation respectively (Fig. 3a). The amplitude of near edge features (7110 to 7160 eV) reduced more than that in the extended edge spectrum (7210 to 7500 eV) within 12 ns. The fast decay of near edge features comparing to the extended edge features indicates that the structural dynamics within 1 ns dominantly originates from the change of electronic states. To quantify the time scale of the induced atomic coordination change, we measured the relaxation kinetics of the photoinduced maximal absorption change at 7123 eV. The characteristic decay time agrees with the strain relaxation as measured by TR-XRD^5^ (Fig. 3b). The matched decay time constant supports the notion that the observed unit cell deformation is correlated with the out-of-plane lattice expansion. Although we cannot exclude the deformation potential mechanism^17^, the observed unit cell deformation are consistent with a photoinduced inverse piezoelectric effect^5^.

### Atomistic Origin of the Spectral Dynamics

**Figure 4:**
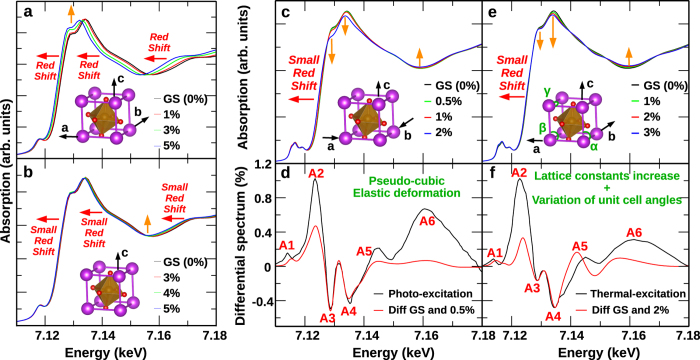
Simulated XAS spectra for structurally distorted BiFeO_3_. Changes in the XANES spectra compared to the undistorted ground state (GS) as a result of (**a**) uniform lattice expansion along *a*,*b*,*c*-axes, (**b**) uniaxial lattice expansion along *c*-axis and (**c**) anisotropic lattice deformation with Poisson’s ratio of 0.34. (**d**) The difference spectrum of (**c**) as a result of optical excitation. (**e**) Changes in the XANES spectrum as a result of lattice constants increase and tilting of the lattice axes. (**f**) The difference spectrum of (**e**) as a result of heating. The black curves in (**d**,**f**) are the experimental data for comparison.

To identify the structural information captured in the experimental absorption spectra, we performed self-consistent, real space density functional theory calculations to simulate photoinduced x-ray absorption spectral changes of BFO thin film in a pseudocubic unit cell representation. The calculated XAS spectrum is based on the ground state BFO structure of the thin film (Supplymentary Materials Note 2). In order to simulate the photoinduced difference spectra, we examined three distorted local structure models (Fig. 4a–c, see Discussion section). Based on the quality of the agreement between the model structures and experimental results, we conclude that an out-of-plane deformation with the Poisson’s ratio of 0.34^18^ is best able to account for the experimental photo-induced XAS evolution. As shown in Fig. 4d, the simulated difference spectrum with 0.5% out-of-plane lattice expansion and 0.17% in-plane lattice compression is in good qualitative agreement with the measured spectrum. This microscopic picture of photoinduced structural effect in BFO advances the understanding of optically driven structural changes from a one-dimensional (out-of-plane) lattice distortion^5^ to a localized three-dimensional lattice distortion.

## Discussion

The theoretical studies of local structural influences on the XANES data qualitatively explain the observed spectrum features upon photoexcitation. We first considered unit cell deformation in two cases: (*i*) a uniform lattice expansion along the *a*, *b*, and *c* axes, imitating a thermal expansion of the pseudocubic lattice, and (*ii*) a uniaxial lattice expansion along the *c*-axis only. The resulting spectra, shown in Fig. 4a,b respectively, clearly highlights that both cases (*i*) and (*ii*) induce a red shift of the whole spectrum. As reported previously^19^, the spectral energy shift is essentially due to a change in the position of the *4p* orbital states of the transition metal atom, which are sensitive to the variation of the Fe-O bond length. Since a global lattice expansion (Fig. 4a) induces a larger increase of the average Fe-O bond length than a uniaxial lattice expansion along the *c*-axis (Fig. 4b), the spectral shift toward lower energies is more pronounced in case (*i*) than in case (*ii*). In addition to the spectral energy shifts, a global lattice expansion also induces an increase of the main edge intensity, as pointed by the orange arrow in Fig. 4a, while for uniaxial expansion along *c*-axis only, the spectrum intensity remains essentially unchanged. Both cases are against experimental observation which clearly shows a decrease in the main edge absorption.

Because the spectral modifications obtained theoretically in these two cases were in poor agreement with the experimental observation, we then investigated how the spectra evolves with an elastic lattice deformation, which implied an expansion along the *c*-axis and simultaneously a compression along the *a* and *b* axes. Although a few unit cells at the interface is epitaxially constrained, the majority of the film is relaxed^20^,^21^ and an in-plane deformation is possible. The simulated spectra for elastic deformation of 0.5%, 1% and 2% along out-of-plane direction, shown in Fig. 4c, present only a small red shift of the absorption edge. The reason for such small shift is that the average Fe-O bond length remains relatively similar to that of the BFO ground state structure, consistent with the Fe *K*-edge EXAFS (Fig. 2b). The small red shift of the main-edge (Fig. 4c) is mainly responsible for the intensity of the A2 peak of the photo-excitation difference spectrum (Fig. 4d). The non-equivalent absorption reduction within the main edge, as shown by the orange arrows, is responsible for having the amplitude of A3 larger than A4 valleys.

Upon photoexcitation, the observed spectroscopic changes can result from both electronic processes and transient temperature increases due to the absorption of radiation energy. To differentiate the electronic contribution from thermal heating, we studied the difference spectra as a result of photoexcitation and heating side by side (Fig. 4d,f). The main discrepancy is the relative amplitude of A3 and A4 valleys: |A3| > |A4| upon optical excitation, while |A3| < |A4| upon heating. To understand this discrepancy, we simulated the heating induced structural changes as a global increase of the lattice constant together with an increase of lattice angles *α* and *γ* and a decrease of *β* by 0.6 degree, which are the results of a geometric distortion with the reduction of the rhombohedral angle upon heating^22^. As shown by the DFT simulation in Fig. 4e, the modification of *α*, *β*, and *γ* can change the relative amplitude effectively. The simulated difference spectrum is in good qualitative agreement with the measured difference spectrum induced by temperature (Fig. 4f). The temperature induced structural change is different from the elastic deformation upon optical excitation because the change occurs adiabatically in thermal equilibrium so that the film has sufficient time to minimize the free energy of the crystal structure by changing lattice angles.

As the modification of *α*, *β*, and *γ* may induce a variation in the FeO_6_ octahedral tilt angles, we next investigated the effect of changes to the oxygen octahedral tilt angle alone. The octahedral tilt angles are important to complex oxide properties^23^,^24^. They are defined by three consecutive oxygen atoms connecting FeO_6_ octahedra (see Supplementary materials Note 5). We found that the octahedral tilt is an effective structural parameter to tune the relative amplitude of A3 and A4 valleys. However, our simulation shows that the reduction of the octahedral tilt upon heating^22^ will lead to an increase of absorption at 7129 eV and a decrease of absorption at 7135 eV (see Figure S6 in Supplementary materials). As a result, the amplitude of the A3 valley increases and the amplitude of the A4 valley decreases, which cannot explain the observed opposite changes that lead to |A3| < |A4| upon heating. Therefore, we conclude that the difference spectra upon heating necessarily involves a modification of the lattice angles, rather than a variation of octahedral tilt.

Another noticeable difference of the photo-induced and heat-induced spectral changes is seen in the intensity of A6 peak respect to A2. The A6 peak is significantly higher upon optical excitation than the case when the sample temperature increases (Fig. 1b). The higher the A6 peak is in the difference spectrum, the shallower the valley at 7160 eV is in the absorption spectrum, indicating stronger damping of the oscillation at the extended x-ray absorption region. The estimation of temperature rise is only about 50 K upon the laser excitation^5^,^7^, which cannot explain the observed large change at 7160 eV due to Debye-Waller effect. In comparison of the difference spectra upon heating, the higher A6 peak intensity indicates inhomogeneous strain. This is consistent with the broadening of the diffraction peak in previous XRD measurements^5^. In contrast, the temperature increase yields homogeneous lattice deformation and the damping of the oscillation is only a result of the Debye-Waller effect. The simulated spectra by DFT calculations cannot capture these changes (Fig. 4e) since the A6 peak results from the interference of x-ray scattering of the first coordinate shell of the Fe atom rather than a change of density of states.

In conclusion, the experimental investigation of a transient structural state of a BFO thin film using TR-XAS with supporting DFT calculations shows that a localized elastic anisotropic lattice deformation captures the structural rearrangement around the Fe atom. This unique light-induced lattice deformation is a result of localized electron-lattice interaction via a piezoelectric distortion, which has been unambiguously shown different from an adiabatic thermally driven lattice expansion. The elastic deformation we document within a BFO unit cell may lead to ultrafast control of magnetism through magnetoelectric coupling in multiferroic materials, opening a new way to manipulate mechanic and magnetic properties with light. The combined x-ray and quantum mechanical methodology to assess the structural dynamics around transition metal atoms upon photoexcitation paves the way to characterize important processes in a wide range of technologically important perovskite complex oxides.

## Methods

### Sample preparation and characterization

The BiFeO_3_ thin films were grown by reactive molecular-beam epitaxy (MBE) on SrTiO_3_ (001) substrates with a miscut less than 0.1 degree^14^. The deposition was conducted in an adsorption-controlled regime utilizing distilled ozone by supplying a bismuth overpressure and utilizing the differential vapor pressures between bismuth oxides and BiFeO_3_ to control stoichiometry. During the deposition, the Fe flux was about 2.0 × 10^13^ atoms/(cm^2^·s) and the Bi flux was 1.1 × 10^14^ atoms/(cm^2^·s). The distilled ozone (~80% ozone) was used to create a background oxidant pressure of 1 × 10^-6^ Torr. The substrate was maintained at a constant temperature of 610 °C.

The structural properties of the sample was characterized by the x-ray diffraction. The measurements of (002)_pc_ and (113)_pc_ Bragg peaks (Supplementary Materials Note 1) show the film is a crystalline thin film. The crystal structure is monoclinic^25^,^26^,^27^, with the remnant polarization points to four possible directions along the <111> axis as expected for a 35 nm-thick film^20^,^21^. The projection of the polarization to the <001> axis gives rise to a depolarization field parallel to the surface normal of the film. At room temperature, the depolarization field is partially screened by the absorbed surface charges in atmosphere. The lattice structure as used in DFT calculation are detailed in Supplementary Materials Note 2.

The electrical properties of the sample was characterized by the resistivity measurement using “Pro4” four-point resistivity systems from Signatone, Co. The resistivity and the sheet resistance of the BFO film are 2.2 ± 0.1 × 10^4^ Ω·cm and (6.3 ± 0.2) × 10^9^ Ω/sq, respectively.

### Time-resolved x-ray absorption measurements

The time-resolved x-ray absorption measurements were carried out at room temperature at 11ID-D of the APS at Argonne National Laboratory (Fig. 1a, inset). The 400 nm, 100 fs pump pulse is the second harmonic output of a fs Ti:Sapphire regenerative amplified laser operating at 10 kHz repetition rate. It is focused to the sample to a 0.7 × 1 mm (FWHM) spot. The x-ray probe pulses with the size of 0.1 × 0.2 mm were derived from electron bunches extracted from the storage ring with 79 ps full-width-half maximum and 6.5 MHz repetition rate. Following the close-to-normal excitation of the laser pulse, an x-ray probe pulse was incident at a 4 degree respect to the sample surface. The x-ray polarization is parallel to the sample surface. Two avalanche photodiodes (APDs), at 90 degree angle on both sides of the incident x-ray beam, were used to collect the x-ray fluorescence signals. A combination of soller slits and Mn filters was inserted between the sample and the APD detector to reduce the background scattering. An identical APD detector positioned in the upstream of the x-ray beam propagation direction from the sample was used to collect air scattering signals as references for intensity normalization.

The outputs of the APDs were sent to a fast analyzer cards (Agilent) triggered by a 10 kHz signal that was synchronized with laser. The card digitized the x-ray fluorescence signals as a function of time at 1 ns per point after each trigger and averaged the signals with 4 second integration time. All x-ray pulses between two laser pulses were recorded. The intensity of each individual x-ray pulse of the sample detector was normalized by the intensity of the same pulse given by the normalization detector. Such a normalization scheme greatly reduced systematic error caused by bunch-to-bunch fluctuations. The fluorescence from the synchronized x-ray pulse at chosen delays after the laser excitation was used for building the excited state spectrum and the ground state spectrum was obtained by averaging x-ray pulses in the previous 20 synchrotron ring cycles.

### DFT calculations

To compute Fe K-edge XANES spectrum of BiFeO_3_, we used self-consistent, real-space x-ray absorption calculations, as implemented in the FDMNES^28^ and FEFF9^29^ codes. In each case, we used a cluster of 8 Å radius, containing 161 atoms, for which the ground state structure used in the calculations is detailed in Supplementary materials (see Figure S1). The final photoexcited state is obtained by solving a Schrödinger-like equation through the Green formalism, within the limit of the muffin-tin approximation. The exchange-correlation was calculated using the real Hedin-Lundquist potentials^30^, as implemented in FDMNES and FEFF9. Spin-orbit coupling, dipoles, quadrupoles and core-hole contributions were taken into account. The spectrum shown in Fig. 4a,b were obtained by FEFF9, while the spectrum shown in Fig. 4c,e were obtained with the FDMNES code.

In order to simulate the experimental resolution and therefore to improve the theoretical and experimental qualitative spectrum agreement, a Gaussian convolution has been added after a Lorentzian convolution, which account for the broadening of the core-hole and spectral width of the final state. In the case of simulated photo-excited spectrum, we found that a slight broadening of the localized 3d states brings some improvements in the description of the difference spectra at near A1 peak region (See Figure S4 in Supplementary materials).

## Additional Information

**How to cite this article**: Wen, H. *et al.* Capturing ultrafast photoinduced local structural distortions of BiFeO_3_. *Sci. Rep.* **5**, 15098; doi: 10.1038/srep15098 (2015).

## Supplementary Material

Supplementary Information
