# Supplementary material for: Capturing ultrafast photoinduced local structural distortions of BiFeO3
Source: Sci Rep. 2015 Oct 14;5:15098. doi: 10.1038/srep15098 (PMC4604520; doi:10.1038/srep15098)
Supplement: Supplementary Information [file srep15098-s1.pdf]

Supplemental Materials for "Capturing ultrafast photoinduced local structural distortion of $BiFeO_3$"

Haidan Wen,[1*] Michel Sassi,[2] Zhenlin Luo,[3] Carolina Adamo,[4] Darrell G. Schlom,[4,5] Kevin M. Rosso,[2§] Xiaoyi Zhang[1†]

[1] X-ray Science Division, Argonne National Laboratory, Argonne, Illinois 60439, USA
[2] Physical Sciences Division, Pacific Northwest National Laboratory, Richland, WA 99354, USA
[3] National Synchrotron Radiation Laboratory, University of Science and Technology of China, Hefei, 230029, China
[4] Department of Materials Science and Engineering, Cornell University, Ithaca, New York 14853, USA
[5] Kavli Institute at Cornell for Nanoscale Science, Ithaca, New York 14853, USA

*wen@aps.anl.gov
§kevin.rosso@pnnl.gov
†xyzhang@aps.anl.gov

1. Static x-ray diffraction of $BiFeO_3$ thin film

The x-ray reflectivity around $BiFeO_3$ (BFO) $(002)_{pc}$ peak shows the film is a crystalline film, evident by clear Kiessig fringes in Fig. S1(a). The splitting of $(113)_{pc}$ Bragg peaks shown in Fig. S1(b) supports that the BFO film has multi-domain structures in which the remnant ferroelectric polarization points to four possible directions along the $<111>_{pc}$-axis (Ref.[26-27]).

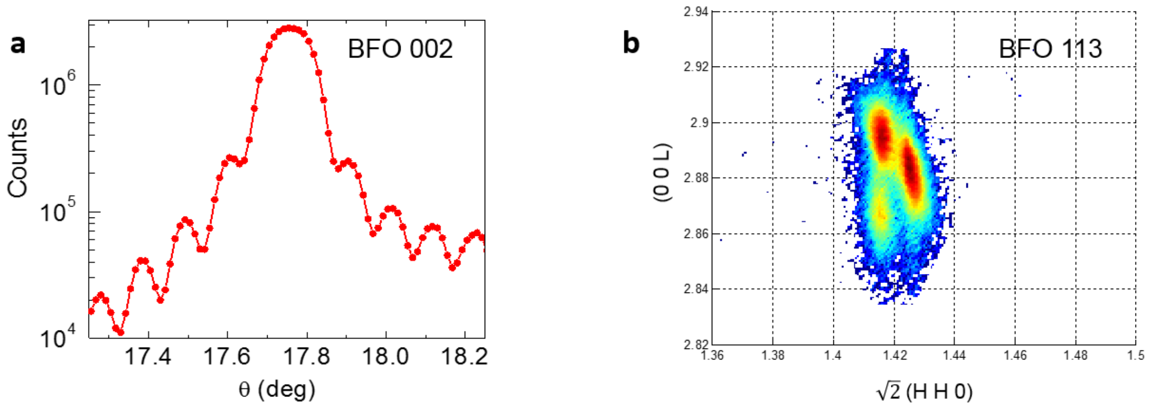

Fig. S1: (a) The radial scan of $(002)_{pc}$ Bragg peak of BFO film. (b) The reciprocal space mapping of $(113)_{pc}$ Bragg peaks in the $(1\bar{1}0)$ plane. The HKL are labeled respect to the $SrTiO_3$ lattice.

2. The structure of the ground state of $BiFeO_3$ as used in the calculations:

For the calculations of x-ray BFO spectrum, we have used a modified version of the bulk structure as referenced by Moreau *et al.* [Ref S1]. The principal modification made to the original bulk BFO structure was on the value of the octahedral tilt angle, θ, defined by O—O—O alignment as shown in Fig. S2(a) and Fig. S2(b). In the original bulk structure, the octahedron tilt is about 10 degrees, while in thin film the octahedron tilt is about 7 degrees [Ref S2]. Based on 7 degree octahedron tilt angle, we have adjusted the lattice parameters of the modified BFO structure in order to keep the Fe—O bond length within each octahedron the same as those in the original structure. Table S1 shows structural characteristics of the original and modified BFO bulk structures. The latter has been used as the reference ground state of the thin film for the calculation of x-ray spectrum shown in Fig. S2(c), which agrees well with the measured ground state spectrum shown in Fig. 1a.

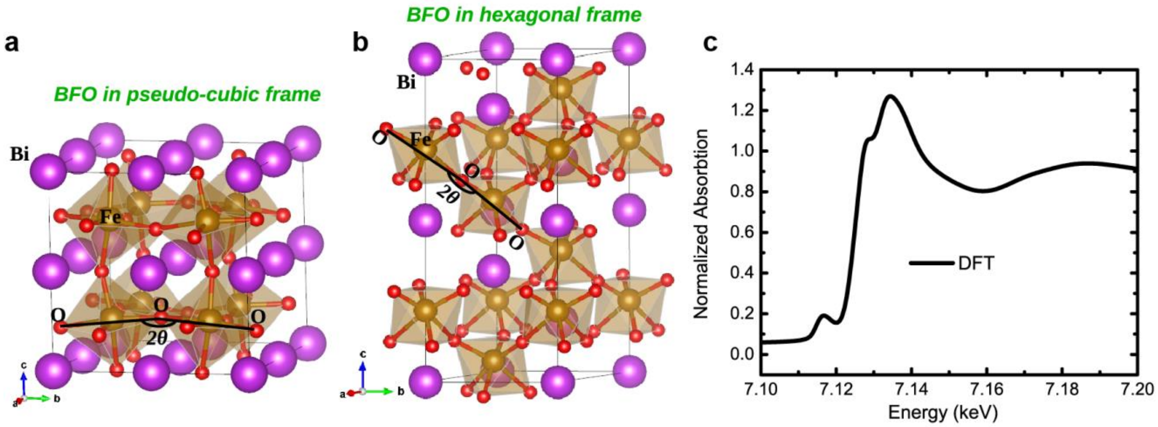

Fig. S2: (a) Pseudo-cubic and (b) hexagonal representation of the BFO ground state structure. (c) The calculated x-ray absorption spectrum based on a modified lattice structure shown in (a).

Hexagonal frame	Original BFO structure [Ref S1]	Modified BFO structure
a=b (Å)	5.5876	5.6446
c (Å)	13.8670	13.9161
Fe—O (x3) (Å)	2.1151	2.1151
Fe—O (x3) (Å)	1.9404	1.9404
Octahedron tilt angle θ (°)	10.06	7.01

Table S1: Some characteristics of the initial and modified bulk BFO structures. The latter has been used as the reference ground state for the calculation of x-ray spectrum.

3. The correspondence between electronic density of state and Fe K-edge XANES spectra.

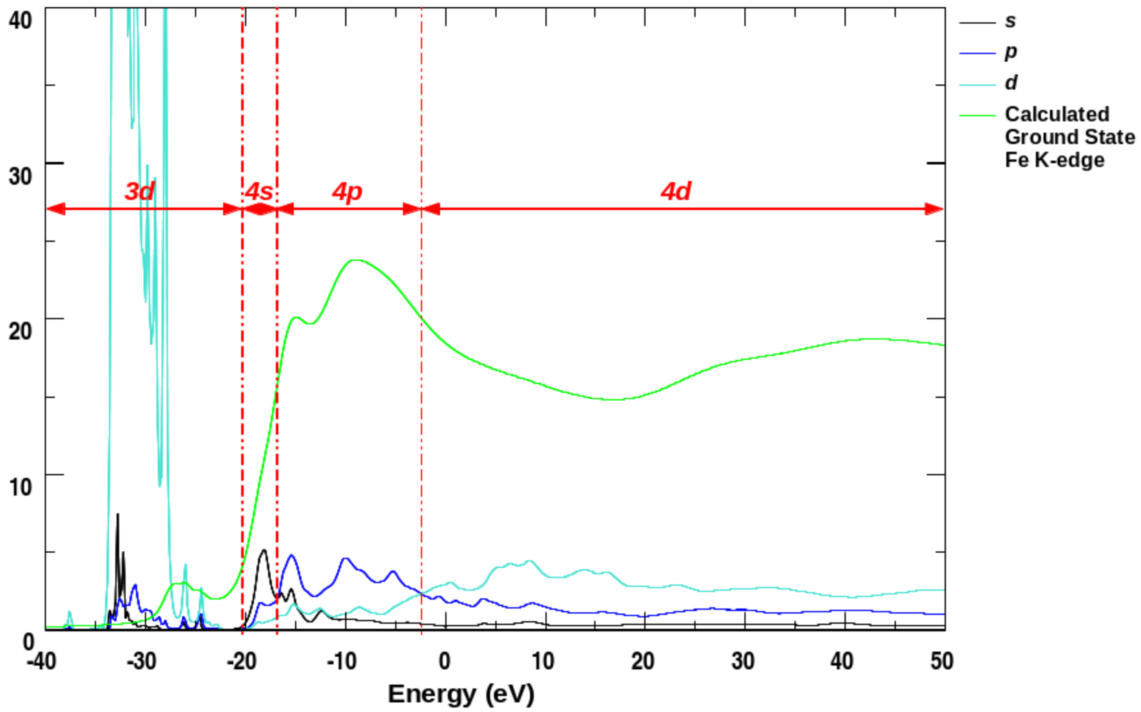

Fig. S3: Correspondence between the Fe K-edge XANES spectra and the density of state projected onto the *s*, *p* and *d* orbitals, as a result of DFT calculation.

4. Effects of an increased broadening of the density of states on the XANES spectra:

In order to improve the qualitative agreement between the calculated and experimental difference spectra, we have tested the effect of a slight uniform increased broadening on the density of states. This effect as a result of temperature increase are mainly responsible for damping the absorption variations by homogeneously reducing (increasing) the intensity of the absorption peak (valley) without introducing any energy shift to the XANES spectra. As shown in Fig. S4, we found that the difference spectra can be improved a bit around the A1 peak region, without significantly modifying the rest of the difference spectra, especially the |A3|/|A4| ratio.

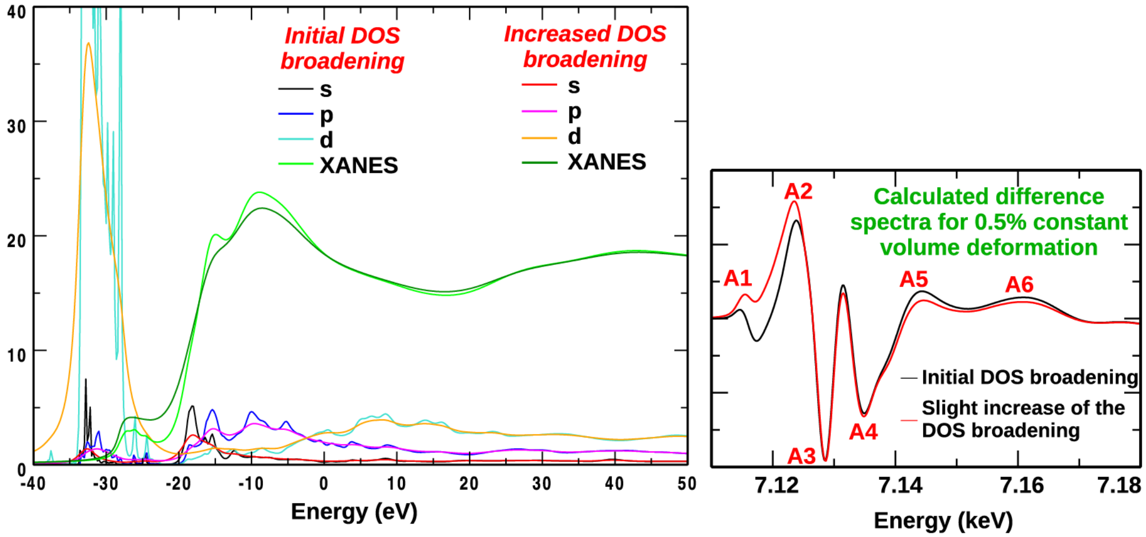

Fig. S4: Effect of increasing the DOS broadening on the XANES and differential spectra. For clarity purpose, we show a plot of the XANES spectra (left panel) with a much larger broadening increase than that used to calculate the difference spectra (right panel).

5. The calculation of octahedron rotation:

To investigate the effect of a variation of the oxygen octahedron tilt angle on the spectral features, we have generated a series of structures from the modified ground state of the BFO pseudo-cubic unit cell. Since the average Fe—O bond length should remain unchanged (Fig 2b), we have assumed each FeO_6 octahedron as rigid octahedron while performing the rotation. This induces a slight volume increase of the pseudo-cubic unit cell, as the octahedron tilt angle reduces. The rotation of the oxygen octahedron around each Fe atom has been done for two cases: (*i*) a rotation within the [110, 001] plane (see Fig. S5), having a four-fold symmetry, and (*ii*) along the [100], [010] and [001] directions simultaneously. These rotations are such that for an octahedron tilt angle of 0 degree, the oxygen octahedrons are aligned along the [001] direction for the case (*i*), while they are aligned along each three [100], [010] and [001] directions for the case (*ii*). In these two cases, the stimulated spectrum as a result of tilt give similar spectral modifications, as shown in Fig. S6 for a series of rotation from 10° to 0°.

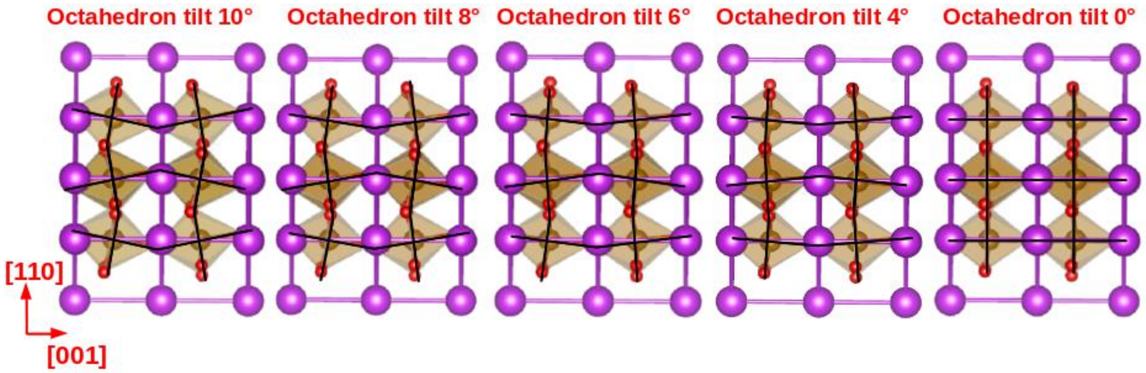

Fig. S5: Structural modifications upon the reduction of the oxygen octahedron tilt angle.

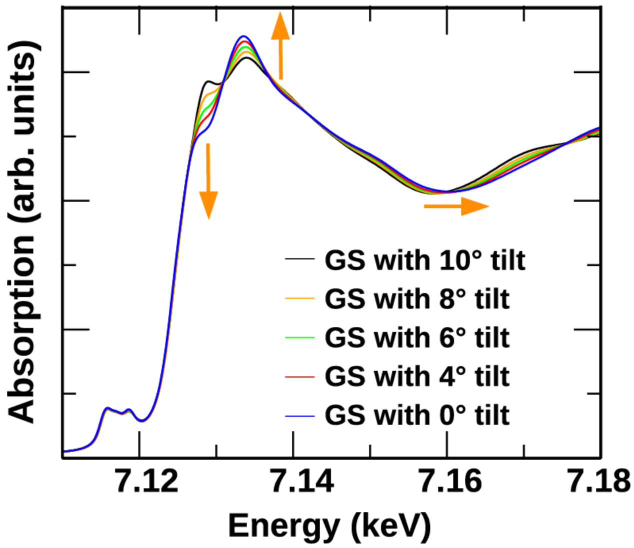

Fig S6: Changes in the Fe K-edge XANES spectra upon the variation of the oxygen octahedron tilt angle in the ground state (GS) structure.

6. The simulation of thermally induced spectrum:

Lattice deformations and atomic reorganizations play a crucial role in modifying the relative peak intensities of the main edge. In particular, the calculations show that keeping the average Fe–O bond length constant is essential to avoid energy shifts of the XANES spectra. If any energy shifts occur, the difference spectrum would be more complicated to calculate and interpret. As shown in Fig.2b, the variation of the average Fe–O bond length obtained experimentally for the thermal excitation case is negligible. All these elements suggest that the cell deformations and atomic reorganizations occurring during thermal excitation are not simple since they must induce an increase of the cell volume, as expected under heating conditions, but also keep relatively constant the average Fe–O bond length. In that regards, finding the exact lattice deformations involved in thermal excitation is a tedious work since many degrees of freedom are available for cell modification upon temperature increase, such as isotropic or non-isotropic variation of length and angles between the lattice vectors and the atomic reorganization in the oxygen octahedrons.

In our study, we simplified the calculation of thermal induced spectra using a constant volume deformation model (*c*-axis expansion and *a, b*-axis contraction) in the hexagonal frame. This simplification is justified by the small temperature rise (250K) that does not yield a significant volume changes (Ref. [22] in the main text). A constant volume deformation in the hexagonal frame results in a global increase of the lattice vectors and a modification of the unit cell angles in the pseudo-cubic frame. This type of cell deformation allows us to include the contributions from lattice and unit cell angle variation (key parameter to switch the A3/A4 ratio compared to the photo-excited case). In this case, constant volume deformation in the hexagonal frame is a good approximation to the much more complicated real case cell deformation with regards to the A3/A4 ratio.

7. Data analysis of EXAFS:

The Athena program is used to process experimental XAS data to extract the normalized oscillation amplitude $\chi^{exp}(k)$ and the photoelectron wave number k is defined by $k = \sqrt{2m(E - E_0)}/\hbar$, where E_0 is the absorption edge energy. The theoretical calculated $\chi^{th}(k)$ is given by EXAFS equation,

$$\chi^{th}(k) = \sum_j \frac{S_0^2 N_j f_j(k)}{kR_j^2} e^{-2k^2\sigma_j^2} e^{-2r_j/\lambda(k)} \sin[2kR_j + \delta_j(k, r_j)]$$

where j indicates a shell with identical backscatters, N_j is the coordination n umber of jth shell, f_j is the backscattering amplitude, R_j is the average distance, σ_j is the mean square variation, δ_j is the scattering phase shift, λ is the effective mean free path and S_0^2 is the amplitude reduction factor.

FEFF6 is used to calculate f_j, δ_j and λ. Fitting to the experimental data to refine the structure parameters S_0^2 , R_j, σ_j^2 is done using the Artemis program. Crystal structure as detailed in Supplemental Materials Note 1 is used as the starting structure for fitting of all spectra. The k^3-weighted Fourier transformed EXAFS spectra with k ranging from 2 to 7.8 Å^{-1} are fitted in R space in the range of 0.8 – 2.2 Å (Fig.S6). Due to the limited k range used in the fitting, only the first Fe-O shell has been fitted. Based on the crystal structure, the first Fe-O shell split into two sets Fe-O_α (1.915 Å) and Fe-O_β (2.072 Å), both with triple-degeneracy. The same σ^2 and distance changes from both paths were used in the fitting.

Four EXAFS spectra were fitted: XTA spectra before (t<0) and after (t=100 ps) laser excitation and two static XA spectra measured at 300 K and 550K. The structural parameters from the fitting

were shown in Table S2. The average Fe-O bond lengths of 4 EXAFS spectra are the same within statistical frame.

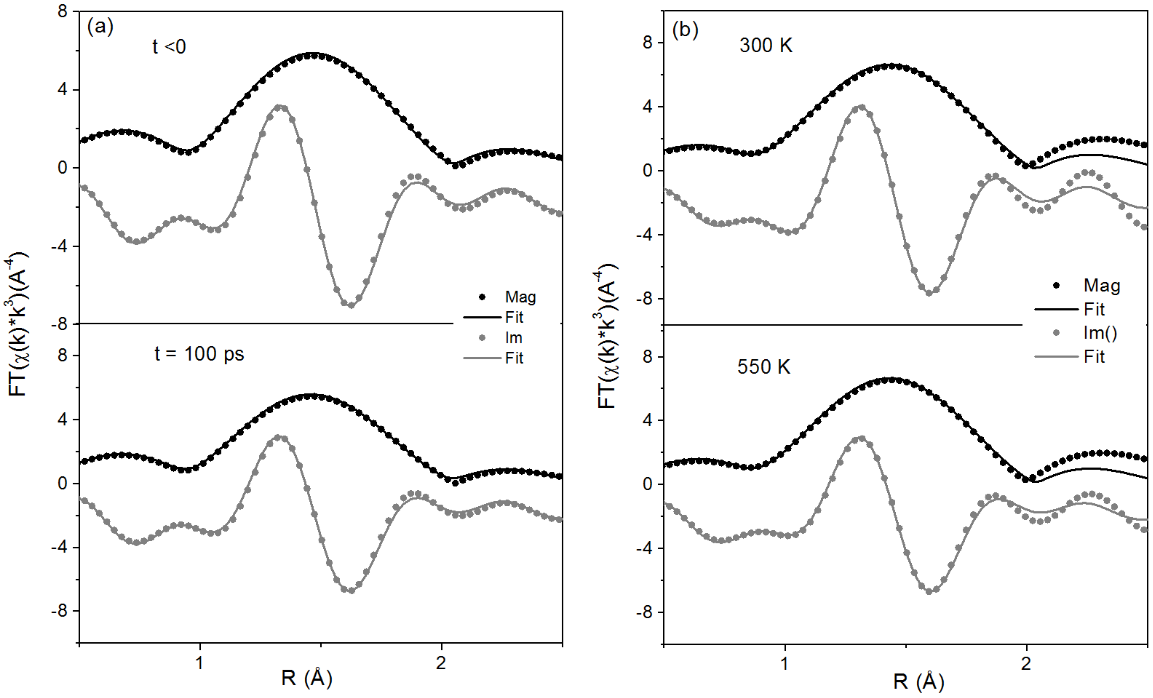

Fig. S7: The Fourier transformed Fe-K edge EXAFS spectra and best fit of (a) XTA spectra before (t<0) and after (t=100 ps) laser excitation; (b) Static XA spectra taken at 300 and 550K respectively. The spectra are phase uncorrected, so the distances R shown in the figure are smaller than the actual best fitting values. The imaginary parts of were offset by -2 for clarity.

	**t<0**	**t=100 ps**	**300 K**	**550 K**
**Fe-Oα (Å) (N=3)**	1.90	1.90	1.89	1.89
**Fe-O_β (Å) (N=3)**	2.05	2.05	2.04	2.04
**σ^2**	0.0033 ± 0.0017	0.0045± 0.0017	0.0013± 0.0021	0.0050± 0.0021

Table S2. Structural parameters extracted from the best fitting.

References for Supplementary materials:

[Ref S1]: J. Moreau, C. Michel, R Gerson and W. James, *J. Phys. Chem. Solids*, **32**, 1315 (1971).

[Ref S2]: A. Y. Borisevich, H. J. Chang, M. Huijben, M. P. Oxley and S. Okamoto, *Phys. Rev. Lett.*, **105**, 087204 (2010).
